# Effects of Mercury Contamination on Microbial Diversity of Different Kinds of Soil

**DOI:** 10.3390/microorganisms10050977

**Published:** 2022-05-07

**Authors:** Xiangqun Zheng, Haoyu Cao, Bo Liu, Man Zhang, Chunxue Zhang, Peizhen Chen, Bo Yang

**Affiliations:** 1 Rural Environment Construction Innovation Centre, Agro-Environmental Protection Institute, Ministry of Agriculture, Tianjin 300191, China; zhengxiangqun@caas.cn (X.Z.); 82101205238@caas.cn (H.C.); 82101185126@caas.cn (B.L.); zhangchunxue@aepi.org.cn (C.Z.); chenpeizhen@caas.cn (P.C.); 2 National Field Science Observation and Research Station of Farmland Ecosystem in Changwu, Institute of Soil and Water Conservation, Northwest A & F University, Xianyang 712100, China; zmcocoo0203@nwsuaf.edu.cn

**Keywords:** soil, mercury, microbial diversity

## Abstract

Soil microorganisms promote the recovery of contaminated soil by influencing the cyclic transformation of various substances. In this study, we investigated the impact of mercury pollution on the structure, composition, and main populations of soil microbial communities using a high-throughput sequencing method and observed that mercury pollution significantly influenced the diversity, structure, and distribution pattern of microbial communities. Furthermore, during mercury pollution, the Shannon and Chao indices decreased for the bacterial communities and increased for the fungal communities. Mercury pollution mainly reduced the relative abundances of Proteobacteria (16.2–30.6%), Actinomycetes (24.7–40.8%), and other dominant bacterial phyla. The relative abundance of Ascomycota decreased by 17.4% and 16.7% in alkaline and neutral soils, respectively, whereas the relative abundance of unclassified_k_Fungi increased by 26.1% and 28.6%, respectively. In acidic soil, Ascomycota increased by 106.3% and unclassified_k_Fungi decreased by 71.2%. The results of redundancy and correlation analyses suggested that soil microbial diversity was significantly correlated with soil properties such as pH, cation exchange capacity, soil organic carbon, and total nitrogen (*p* < 0.05) under different treatments. Our findings highlight the impact of Hg pollution on soil microbial communities, thereby providing a theoretical foundation for the bioremediation of soil Hg pollution.

## 1. Introduction

Mercury (Hg) is considered to be one of the toxic metals that affect human health due to its volatility, persistence, and bioaccumulation [1]. With societal development, industrial applications are also in high demand for mercury (Hg), which increases the threat to biological health caused by mercury [2]. At low levels, Hg exposure can damage neurocognitive functions, such as fine motor skills, speech function, memory, and concentration, and can enter the human body through epidermal absorption, respiratory inhalation, and digestive tract intake [3]. Currently, soil pollution by mercury is the focus of most research, as the basis of agricultural production, soil quality, and safety are of significant importance. Most of the mercury in soil is caused by industrial emissions [4], mercury emissions to the atmosphere, and wet and dry deposition leading to mercury pollution in the soil system [5]. The different forms of Hg in soil and the complex soil structure make Hg pollution treatment more difficult [6]. At present, approximately 1.6% of farmland soil in China has been polluted by mercury [7], which directly affects the quality and yield of agricultural products and irreversibly damages the soil environment [8]. Soil microbial communities are considered to be early warning and sensitive indicators of changes in the soil environment and play a key role in managing soil ecosystems [9]. Microorganisms are indispensable components of soil [10]. Microorganisms play an important role in enhancing soil fertility [11], actively participating in the entire process of soil from production to maturity [12], promoting the circulation of C, P, N, S, and other elements in the environment, purifying pollutants, and regulating the balance of the ecosystem [13]. Soil and microorganisms complement each other, and soil provides nutrients to promote the reproduction and growth of microorganisms. The stress of heavy metals usually causes many chemical, physiological, and morphological changes in microbial systems and changes in the metabolic activities of microorganisms, such as protein synthesis [14]. The growth of microorganisms, such as soil fungi and bacteria, is inhibited by mercury contamination of soil, and their life activities and community structure are seriously affected. The Nitrospira phylum is less active upon chronic exposure to high concentrations of mercury, and fungi tend to develop resistance to high concentrations of mercury [15,16]. Several studies have found that the combined pollution of heavy metals, cadmium, and mercury reduces microbial diversity [17,18]. Frossard et al. [19] studied the effects of different mercury concentrations on soil microbial communities and found that the community structure and composition of soil bacteria and fungi were seriously lost and changed in diversity at a mercury concentration of 32 μg g^−1^. At the same time fungal communities are generally less affected than bacterial communities are. Most researchers believe that mercury pollution decreases soil microbial diversity and alters the microbial community structure [20,21]. However, recent studies have observed an increase in soil bacterial diversity with long-term Hg contamination [22]. In soil ecosystems, fungi are more tolerant to heavy metals than bacteria [23,24], and some scholars have studied the relationship between soil microorganisms, heavy metal pollution, and soil physicochemical properties, and found that the relationship between soil microorganisms and soil physicochemical properties is stronger than that of heavy metals [25].

When exogenous mercury enters the soil, it undergoes a series of interactions, such as complexation, surface adsorption, exchange reactions, chelation, and precipitation, which reduces the biological effectiveness, that is, the aging effect [25]. The soil microbiome can simultaneously tolerate heavy metals through regulatory transport, separation, and production of siderophores as it gradually adapts to Hg stress [26]. In some bacterial genomes, the operational system with the core gene merA/merB, which encodes detoxification proteins, is a known defense system against Hg [27]. The soil microbiome increases its resistance to these two genes, which influences several bacteria, such as *Escherichia coli*, *Proteus*, and *Bacillus* [28], to develop tolerance to Hg stress. Dash and Das [27] also showed that the proportion of bacteria that resist mercury in the soil microbiome is directly proportional to the level of mercury pollution in the environment.

The tolerance of soil bacterial communities mainly depends on Hg solubility in the soil [19], which is directly influenced by the physical and chemical properties of the soil [29]. Pollutant stress can cause microbial populations to decrease and even die or develop resistance to become the dominant community [30,31]. The microbial community diversity changes with heavy metals [32], the relative abundance of strongly tolerant microorganisms will increase, and the relative abundance of sensitive microorganisms will decrease. Simultaneously, microorganisms change the number and activity of enzymes, and the soil ecosystem tends to be polluted [33]. However, the impact of exogenous Hg pollution on soil bacterial communities with different properties remains unclear. Based on Illumina MiSeq high-throughput sequencing technology, we analyzed the changes in soil bacterial diversity and community structure characteristics with exogenous Hg after 180 days of aging, which aimed to clarify the impact of Hg pollution on soil bacterial diversity and community structure with different properties. These results are expected to provide a scientific basis for the risk assessment and treatment of Hg-contaminated soils.

## 2. Materials and Methods

### 2.1. Test Setup

The pot experiment was performed in a greenhouse (39°5′49″ N, 117°8′47″ E) at the Environmental Protection Research and Monitoring Institute of the Ministry of Agriculture and Rural Affairs, Tianjin. The test soils considered were the Shaanxi loess soil (S), Hunan paddy soil (H), and Guangdong red soil (G). The soil physical and chemical indices were determined according to conventional methods at the end of the mercury aging experiment [34], and the physical and chemical properties of each soil are shown in Table 1. Air-dried and sieved uncontaminated soil (5 kg) was added to each test pot made of PVC material (height 0.6 m, diameter: 0.2 m). Two types of treatments were considered for each soil in this experiment: (1) control treatment without exogenous Hg (SC, GC, and HC); (2) treatment with containing 2.0 mg kg^−1^ Hg (NO_3_)_2_ solution (SH, GH, and HH). The treatment mixtures were stirred evenly and aged at room temperature for 180 days. Each treatment was repeated three times.

### 2.2. Extraction, Sequencing and Processing of Soil Microbial DNA

Approximately 0.5 g of soil was weighed before adding exogenous Hg and after aging. Thereafter, DNA was extracted using a Fast DNA SPIN Kit according to the manufacturer’s instructions. The extracted soil DNA was subsequently amplified by PCR, using universal primers with different TAG tags. The cut gel was purified using a NanoDrop^®^ (Thermo Fisher Scientific, Waltham, MA, USA). The DNA concentration was determined using an ND-2000 UV-Vis spectrophotometer (Thermo Fisher Scientific, Waltham, MA, USA), and the purified DNA was sequenced using a MiSeq PE 300 sequencer (Illumina Inc., San Diego, CA, USA) from Shanghai Meiji Biomedical Technology Co., Ltd. (Shanghai, China) After sequencing, low-quality sequences were removed, effective sequences were distinguished, and the sequence direction was adjusted according to the barcode and primer sequences.

### 2.3. Data Statistics and Analysis

Species composition, sample comparison, and correlation between environmental factors and bacterial communities were performed on a cloud platform developed by Shanghai Meiji Biomedical Technology Co., Ltd. The Operational Taxonomy Units (OTUs) were classified using Usearch v7.0 (http://www.drive5.com/usearch/, 23 January 2020), with a 97% sequence similarity threshold. The Shannon index was used the observed OTU to describe the alpha diversity of each sample and to compare the level of bacterial diversity. Principal coordinate analysis (PCoA) was used based on the distance between Bray and Curtis, and the “labdsv” software (R_4.0.2, The University of Auckland, Auckland, New Zealand) package was used to describe the β-diversity of the bacterial community.

## 3. Results

### 3.1. Differences in Soil Properties

**Figure 1:**
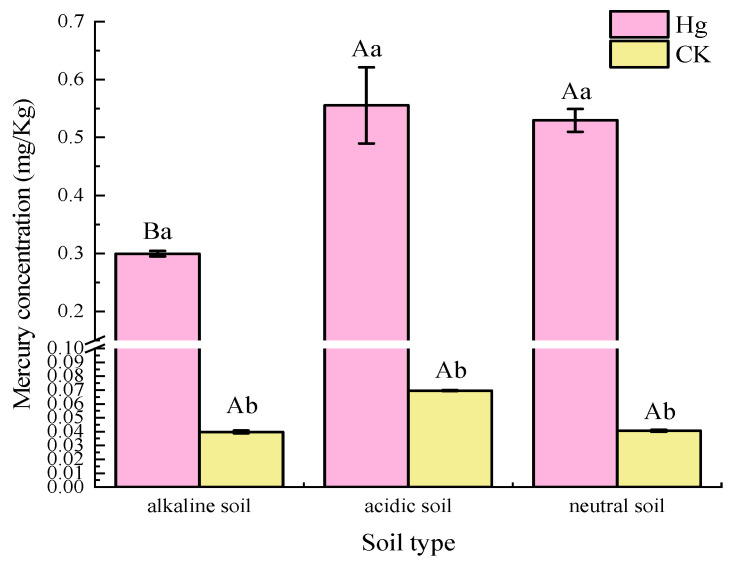
Changes of mercury content in three soils after 180 days of aging. Capital letters in the figure indicate significant differences between soil groups, and lowercase letters indicate significant differences within groups: *p* < 0.05.

Acidic soil was better than that of the other two soils. The background Hg was at similar levels in the acidic soils of Guangdong and the neutral soils of Hubei. After the addition of exogenous mercury, the mercury content of the alkaline soil in Shaanxi was significantly lower than that of the other two soils (*p* < 0.05) (Figure 1). Soil physicochemical properties were not significantly different between soil types after adding Hg from an external source; only the acidic soil pH content was significantly (*p* < 0.05) increased (Table 1).

### 3.2. The Impact of Mercury Pollution on the Diversity of Soil Bacteria and Fungi with Different Properties

**Figure 2:**
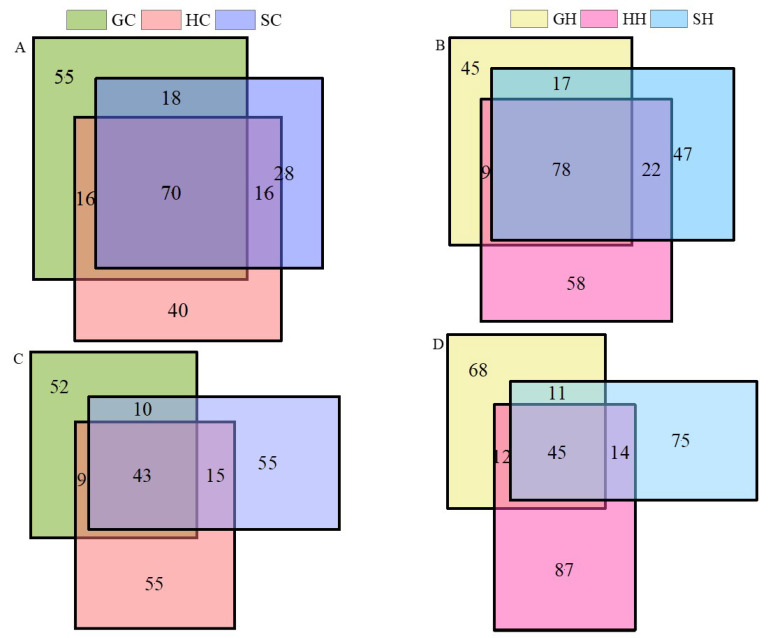
The Venn diagram shows common or unique OTUs number in the different treatments in three soils with different pH. Figure (**A**,**B**) shows the changes in bacterial OTUs before and after the addition of exogenous mercury, respectively, and Figure (**C**,**D**) shows the changes in fungal OTUs affected by mercury.

**Figure 3:**
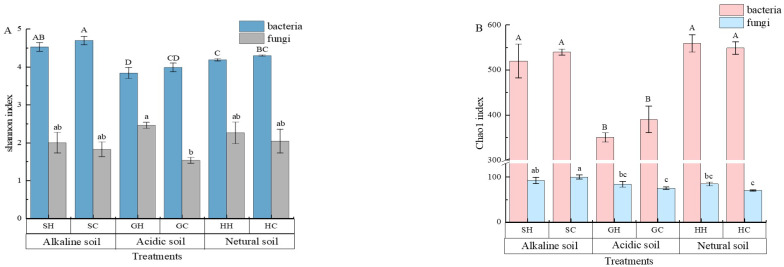

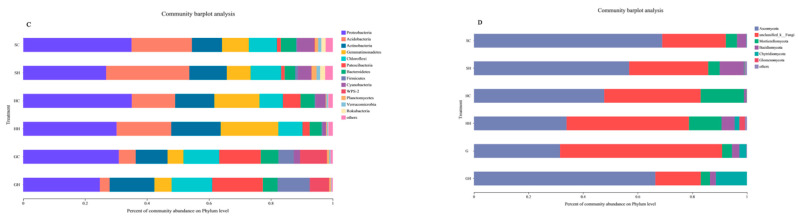
The shannon and chao indexes of the microbial community in soils with three different acidity levels (**A**,**B**); Predominant flora species in relative abundance at the microbial phylum level of three soil treatments. (**C**,**D**) represent bacteria and fungi, respectively. Capital letters in the figure indicate significant differences in bacterial communities among groups and lowercase letters indicate significant differences in fungal communities among groups (*p* < 0.05).

**Figure 4:**
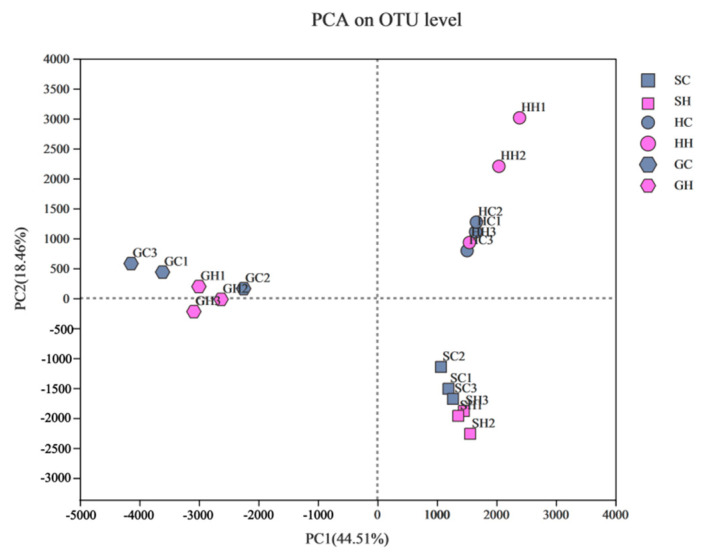
Principal component analysis (PCA) of bacterial communities at the phylum level for all samples. Alkaline soils are represented by triangles; neutral soil samples are represented by squares; acid soil samples are represented by circles, mercury treatments are filled in pink; control treatments are filled in green.

After optimizing and filtering the low-quality sequences, the average number of sequences retained per sample was 5630. Furthermore, the coverage index of each sample was above 98%, indicating a possible high rate of species detection, and the sequencing results suitably included the bacterial communities in the test soil. In terms of bacteria as shown in Figure 2A,B, the OTUs increased among the three soil types (from 70 to 78) after being affected by exogenous mercury. The OTUs that were unique to neutral soils increased from 40 to 58 and alkaline soils increased from 28 to 47, but the OTUs specific to acidic soils decreased from 55 to 45. From the fungal samples (Figure 2C,D), the OTUs unique to GC, HC, and SC were 52, 55, and 55, respectively, and increased to 68, 87, and 75 after treatment with exogenous mercury, and shared by the three soils only increased by 2 OTUs to 45. As shown in Figure 3A, one-way analysis of variance (Chao index and Shannon index) of soil microorganisms at the genus level in the three soils before and after Hg pollution showed that the bacterial diversity of alkaline soil (Shaanxi) was significantly higher than that of neutral soil (Hubei) and acidic soil (Guangdong) before mercury pollution (*p* < 0.05). After mercury pollution, the soil Shannon index decreased, but alkaline soils were still significantly higher than neutral soil and acid soil (*p* < 0.05). The Chao index of alkaline and neutral soils before and after mercury pollution was significantly higher than that of acidic soils (*p* < 0.05). The Shannon and Chao1 indices of alkaline and acidic soils showed a decreasing (albeit non-significant) trend after mercury pollution, and the Shannon diversity indices were statistically significant in all three soil types (*p* < 0.05), indicating that mercury pollution can reduce the density and diversity of soil bacteria. PCA (Principal Component Analysis) based on the Bray–Curtis distance algorithm on the OTU levels of the bacterial communities in the three soils before and after mercury pollution showed that the community differences were significant in the three soil bacterial communities, which were completely separated and at a distance from each other before and after mercury pollution (Figure 4). In terms of soil types, acidic soils are clearly separated from alkaline soils and neutral soils on PC1, and alkaline soils are clearly separated from neutral soils on PC2. The composition of bacterial communities showed dramatic variations among the three soil types; biodiversity was greatest in alkaline soils and least in acidic soils.

**Figure 5:**
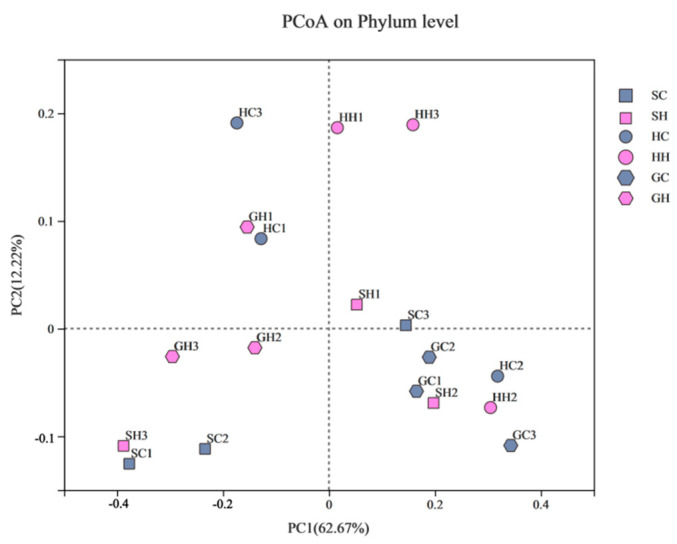
Principal co-ordinates analysis (PCoA) of fungal communities at the phylum level for all samples. Note: Alkaline soils are represented by triangles; neutral soil samples are represented by squares; acid soil samples are represented by circles, mercury treatments are filled in pink; control treatments are filled in green.

As shown in Figure 3B, before mercury pollution, no significant difference was observed in fungal community diversity in the three soils; however, the abundance of fungal communities in alkaline soils was significantly higher than that in the other two soils (*p* < 0.05). The diversity and richness of fungal communities in neutral and acidic soils showed an upward trend after mercury pollution. The Chao index of neutral soil and Shannon index of acidic soils reached statistically significant levels (*p* < 0.05). Principal coordinate analysis (PCoA) reflects the similarities and differences in the fungal community composition before and after mercury pollution in the three soils. In this study, PC1 and PC2 were the two most distinct characteristics that caused sample differences, explaining 74.89% of the fungal community variations (Figure 5). The differences of GC and GH were significant, and they were located on the positive and negative axes of PC1; from the perspective of the PC2 axis, there was no significant difference in the distribution of HH and HC.

### 3.3. The Impact of Mercury Pollution on the Composition of Soil Microbial Communities with Different Properties

In total, 13 taxa were obtained at the phylum level, and bacteria with a relative abundance of less than 1%, with no annotation at this level, were classified as others. Although the three soils contained similar main phyla, their abundances differed significantly. For bacteria (Figure 3C), the five dominant phyla in alkaline and neutral soils were Proteobacteria (35.0% and 35.1%), Acidobacteria (19.5% and 14.0%), Actinobacteria (9.7% and 12.6%), Gemmatimonadetes (8.7% and 14.6%), and Chloroflexi (9.0% and 7.5%), respectively, which accounted for more than 80% of the relative abundance of bacterial communities (Figure 3C). The top five dominant phyla in the soil were Proteobacteria (30.9%), Actinobacteria (10.3%), Chloroflexi (11.5%), Patescibacteria (13.4%), and Firmicutes (5.7%). Although the dominant bacterial phyla remained unchanged before and after Hg pollution in the three soils, their abundance varied. In general, the relative abundance of the phylum Proteobacteria decreased (16.2–30.6%), while the relative abundance of the other dominant bacterial phyla mostly observed an upward trend. The proportion of phyla with relative abundances of less than 5%, such as Bacteroidetes, Planctomycetes, and Verrucomicrobia, further decreased, which is consistent with the results of the Shannon and Chao index analyses.

At the fungi phylum level, Ascomycota, unclassified_k__Fungi, Mortierellomycota, Basidiomycota, Chytridiomycota, and Glomeromycota were found in all three soils (Figure 3D). After mercury pollution, the relative abundance of Ascomycota in both alkaline and neutral soils decreased to 17.4% and 16.7%, respectively, whereas the relative abundance of unclassified_k__Fungi phyla increased by 26.1% and 28.6%, respectively. The dominant fungi in acidic soils, with considerable variations in the relative abundance of the phyla, were Ascomycota, which increased by 106.3%, and the unclassified_k__Fungi phyla, which decreased by 71.2%. The relative abundance of Mortierella in the neutral soil was the only one higher than 10% among the three soils. Basidiomycota in alkaline and neutral soils increased by 161.8% and 500.0% after mercury pollution, respectively, while they decreased in acidic soils by 18.5%, and chytrid in acidic soils increased by 338.5% after mercury pollution.

### 3.4. The Relationship between Soil Environmental Factors and Microorganisms

**Figure 6:**
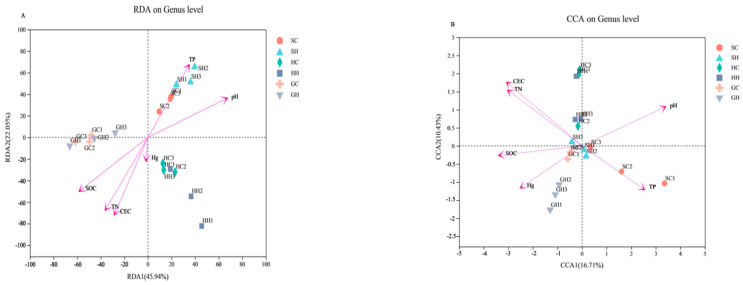
Redundancy analysis revealed the correlations of soil properties and heavy metals at the sample. (**A**) bacteria; (**B**) fungi.

As shown in Figure 6A, at the genus level, soil SOC content and pH considerably affected the soil bacterial community. The SOC content was significantly negatively correlated (*p* = 0.001), whereas the pH value was significantly positively correlated (*p* = 0.001); soil CEC value (*p* = 0.001), TP content, and TN content had significant effects on soil bacterial communities. Among them, the soil CEC value (*p* = 0.001) and TN content (*p* = 0.001) were significantly negatively correlated, and the TP content (*p* = 0.001) exhibited a significant positive correlation. The Hg content had no effect on soil bacterial communities (*p* = 0.586). Soil fungal communities were significantly positively correlated with pH (*p* = 0.005), while CEC (*p* = 0.01), SOC (*p* = 0.012), and TN (*p* = 0.02) were significantly negatively correlated with fungal communities. As with bacteria, the effect of Hg on fungal communities remained insignificant, and the TP content had no effect on soil fungal communities (Figure 6B).

**Figure 7:**
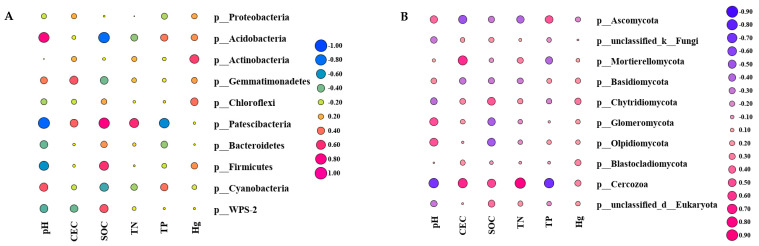
Pearson correlation analysis between the relative abundance of dominant soil microbial phyla and soil physicochemical and mercury content among different treatments. The value of the Pearson correlation coefficient is represented by the color of each circle. Pink indicates positive correlation; blue indicates negative correlation: (**A**) bacteria; (**B**) fungi.

The relationship between soil physicochemical properties and the relative abundances of the top 10 dominant soil microorganisms was studied using Pearson correlation analysis at the phylum level in the three soils. The intensity of the colors and size of the circles are proportional to the Pearson correlation coefficient. As shown in Figure 7A, Pearson correlation analysis demonstrated that Proteobacteria had no significant correlation with changes in the soil physicochemical environment, as the phylum with the highest relative abundance (Figure 3C). At the phylum level, Acidobacteria and Cyanobacteria were significantly positively correlated with soil pH. Patescibacteria and Firmicutes were significantly negatively correlated with soil pH. Patescibacteria and soil TP content were significantly negatively correlated with soil pH. Patescibacteria, Firmicutes, and WPS-2 were positively correlated with SOC. Acidobacteria and cyanobacteria were negatively correlated with soil SOC, Actinomycetes were significantly positively correlated with soil Hg content, Blastomonas and soil CEC content were significantly positively correlated, and Patescibacteria phylum and soil TN content were significantly positively correlated. The effects of Hg on soil bacteria were mostly positive, but mostly insignificant. Among them, Actinobacteria only had a significant positive correlation with mercury content and little correlation with other soil environmental factors.

For fungi, Cercozoa was highly sensitive to the soil environment, was significantly negatively correlated with pH and TP (*p* < 0.05), and significantly positively correlated with SOC, CEC, and TN, with TN reaching a highly significant level (*p* < 0.001). The changes in CEC and Mortierellomycota were negatively correlated. The phyla Glomeromycota and Olpidiomycota were positively correlated with pH, but significantly negatively correlated with SOC (*p* < 0.05). In addition, the top ten fungi species at the phylum level showed no statistical correlation with the soil Hg content (Figure 7B).

## 4. Discussion

### 4.1. Response of Soil Bacterial Diversity and Community Structure to Mercury Pollution

Soil microbial diversity and community structure are affected by various soil environmental variables [35]. Mercury pollution can be an important driver of soil microbial diversity, indirectly affecting bacterial diversity by affecting soil properties [36,37]. In general, existing results suggest that, on a large geographic scale, soil type is one of the most important factors affecting the composition of soil microbial communities [38]. Heavy metals affect soil properties and bacterial community structures differently in acidic and neutral soils. Under the influence of Hg, the bacterial abundance and diversity decreased slightly (Figure 3A,B) in this study. Our results were similar to those of Freyb et al. [15]. This corroborates the influence of the mercury-mediated soil bacterial community structure and life activities. As shown in Figure 7A, the composition of the soil bacterial community is closely related to the acidity and alkalinity of the soil [39]. In this study, the status of bacterial communities at the phylum level in alkaline and neutral soils significantly differed from that in acidic soils, indicating that soil acidification can explain the significant changes in the soil bacterial community structure [40]. The relative abundance of Proteobacteria, Actinomycota, Acidobacteria, and Blastomonas was higher in the three soils, which is consistent with a previous report, suggesting that these phyla were the main bacteria in soil [41,42], with strong environmental adaptability [43]. The relative abundance of Actinobacteria increased with elevated mercury pollution, while the relative abundance of Proteobacteria was negatively correlated with Hg, but overall, it was still the top three dominant bacterial phyla, indicating that both are predominant in mineral sandy soils with poor water and nutrient supply and relatively low pH [44]. Under heavy metal pollution conditions, the abundance of some bacteria increased, whereas the abundance of others decreased because of their tolerance to heavy metals. In contrast, SOC is also an important factor affecting microbial community structure, which is consistent with previous studies [45]. In this experiment, the relative abundance of Acidobacteria was negatively correlated with the soil SOC content. The relative abundance of Acidobacteria was decreased in acidic soils compared to alkaline and neutral soils, and the relative abundance of Acidobacteria in GH was significantly lower than that in GC, contrary to the study by Lauber et al. on the predominance of Acidobacteria in strongly acidic soils [40]. This was because Acidobacteria belong to the oligotrophic group [46], and the relative abundance of Acidobacteria in acidic soils is shown in Figure 3C. The degree was significantly lower than that of the alkaline and neutral soils, and the organic matter of acidic soils was as high as 46.09 g/kg, which was significantly higher than that of the other two soils (*p* < 0.05), which was also well confirmed.

Existing studies on the effect of Hg pollution on soil microbial activity are lacking, indicating that Hg may not be a key factor in the inhibition of soil microbial functions [22]. In this study, there was no significant occurrence of bacterial communities in the three soils before and after mercury pollution. These changes confirm this conjecture. This may be explained by the following reasons: (1) the bacteria in the soil live on biofilms, or live in micro-locations on the surface of organic matter, or are wrapped by micro-aggregates [47], which may prevent them from being exposed to the toxic effects of mercury pollution; (2) after 180 days of aging, mercury and soil colloids reach equilibrium [48], and native soil bacteria adapt to the soil Hg concentration; and (3) some major soil bacteria are relatively insensitive to heavy metals [49]. 

### 4.2. Response of Soil Fungal Diversity and Community Structure to Mercury Pollution

Compared with bacteria, fungi have a larger biomass and lower environmental requirements. Extracellular polysaccharides on the surface of many eukaryotic microorganisms (such as algae and fungi) are negatively charged and can act as adsorbents to prevent the entry of heavy metal ions into cells [50]. Certain fungi have an intracellular heavy metal detoxification mechanism, that is, they accumulate heavy metal ions in special organelles. Once metal ions enter the cell, they can be converted into less toxic compounds [51]. In general, only high concentrations of Hg pollution can affect the survival of soil fungi (such as growth inhibition, cell biochemical inhibition, and morphological destruction), thereby inhibiting the diversity of fungal communities in the soil and resulting in a decline in community diversity and richness [52]. Some scholars have shown through study indicates cultivated soil in acidic areas that pH was the predictor of the bioavailability of heavy metals to effect, leading to changes in the absolute abundances of sensitive fungi in heavy metal-contaminated soils [53]. In this study, we found that changing pH altered soil fungal communities after mercury pollution, and shifts in the soil fungal communities in alkaline soils resembled those in neutral soils and the opposite in acidic soils. In this study, the abundance of Ascomycota and Chytridomycota in acidic soils increased significantly due to mercury pollution (by 106.3% and 338.5%, respectively), indicating their dominance as mercury-tolerant fungi in acidic soils, whereas Basidiomycota in alkaline and neutral soils increased by 161.8% and 500.0% after mercury pollution, indicating its dominance as mercury-tolerant fungi in alkaline and neutral soils. This study suggests that high N addition usually decreases soil microbial biomass and microbial diversity [35,54]. As the variation found in soil properties varied, soil microorganisms changed in terms of their diversity and community assembly. The SOC content of acidic soils was significantly higher than that of alkaline and neutral soils, which may explain why the fungal community changes were different between acidic, neutral, and alkaline soils. 

In contrast to Lin et al. [55], our results could be attributed to the fact that the tolerance of these fungi to mercury in the short term is higher than that of metals such as lead, cadmium, and zinc, so that the fungi have sufficient ability to resist the pressure of mercury and ultimately have no negative impact on biodiversity [56]. Moreover, the adsorption of Hg^2+^ by mineral colloids in the soil may weaken the effect of Hg on soil microorganisms. 

## 5. Conclusions

The entry of heavy metal mercury into soil has a serious impact on the structure and function of the soil microbial community phyla. Changes in soil microorganisms under Hg contamination were studied in three soils with different pH levels, and differences were found in the effects of Hg on different types of soil. Hg input caused changes in the abundance and diversity of soil bacteria and fungi. Bacterial diversity and abundance decreased in all three soil types, fungal diversity increased significantly in acidic soils, fungal abundance increased significantly in neutral soils, and the community structure of microorganisms also changed as a result of mercury. Proteobacteria in the three soils were more sensitive and tended to die after mercury input. Acidobacteria and Actinobacteria were positively correlated with Hg, and Firmicutes were more tolerant to Hg in acidic soils than in alkaline and neutral soils. Soil organic matter was strongly correlated with soil acidity and alkalinity, with acidic and neutral soils being significantly positively correlated with it, while the opposite was true for alkaline soils. Total phosphorus made the greatest positive contribution to changes in microbial community structure, and mercury was a major factor affecting microbial communities in both neutral and acidic soils. In conclusion, metallic Hg and soil acidity together influence the community structure of soil microorganisms. At present, the scope and depth of research on the overall environmental impact of Hg on soil is insufficient. In the future, the mechanisms of microbial responses to soil environmental factors following Hg contamination should be further investigated.

## Figures and Tables

**Table 1:** Soil properties at each soil with different treatments. Abbreviations: TN, total nitrogen; TP, total phosphorus.

		Chemical Properties				
Soil Types	Treatments	pH	CEC	SOC	TN	TP
Alkaline soil	SH	8.347 ± 0.088Aa	10.520 ± 0.058Ca	13.373 ± 0.204Ca	0.989 ± 0.039Ba	0.931 ± 0.024Aa
	SC	8.320 ± 0.153Aa	10.533 ± 0.233Ca	14.510 ± 0.196Ca	1.000 ± 0.0377Ba	0.893 ± 0.043Aa
Acidic soil	GH	4.950 ± 0.153Ca	19.213 ± 0.095Ba	44.970 ± 0.950Aa	1.927 ± 0.0241Aa	0.563 ± 0.021Ba
	GC	4.880 ± 0.116Db	19.247 ± 0.419Ba	44.937 ± 0.825Aa	2.030 ± 0.0.022Aa	0.550 ± 0.018Ba
Neutral soil	HH	6.783 ± 0.088Ba	20.677 ± 0.514Aa	33.890 ± 0.215Ba	2.001 ± 0.014Aa	0.524 ± 0.002Ba
	HC	6.813 ± 0.088Ba	20.270 ± 0.158Aa	33.667 ± 0.665Ba	2.009 ± 0.044Aa	0.572 ± 0.012Ba

Note: Different capital letters within the same column indicate significant differences between different treatments of three soil types at *p* < 0.05, as determined by Student’s multiple range tests; different lowercase letters within the same item indicate significant differences between chemical indices at *p* < 0.05, as determined by Student’s multiple range tests.

## Data Availability

Not applicable.
